# Combination therapy with lansoprazole and cholecalciferol is associated with a slower decline in residual beta-cell function and lower insulin requirements in children with recent onset type 1 diabetes: results of a pilot study

**DOI:** 10.31744/einstein_journal/2022AO0149

**Published:** 2022-11-09

**Authors:** Raghunatha Reddy, Devi Dayal, Naresh Sachdeva, Savita Verma Attri, Vinod Kumar Gupta

**Affiliations:** 1 Postgraduate Institute of Medical Education and Research Chandigarh India Postgraduate Institute of Medical Education and Research, Chandigarh, India.

**Keywords:** *Diabetes mellitus*, type 1, Blood glucose, Glycated hemoglobin A, Fasting, C-peptide, Drug tapering, Glycemic control, Cholecalciferol, Lansoprazole

## Abstract

**Objective:**

To investigate the effects of combination therapy with cholecalciferol and lansoprazole on residual β-cell function and glycemic control in children with new-onset type 1 diabetes.

**Methods:**

Children aged 6-12 years with type 1 diabetes were allocated to receive cholecalciferol and lansoprazole (Group 1) or no treatment (Group 2). Children were maintained on their respective insulin regimens and kept records of blood sugar and insulin doses taken. Children were followed at three-month intervals for six months. Changes in mean fasting C-peptide and HbA1c levels, daily insulin doses, fasting blood glucose and mean blood glucose levels from baseline to end of the study were analyzed.

**Results:**

Twenty-eight children (14 per group) met the eligibility criteria. Fasting C-peptide levels decreased significantly from baseline to study end in both groups (mean decrease -0.19±0.09ng/mL and -0.28±0.08ng/mL, p=0.04 and p=0.001; Group 1 and Group 2 respectively). However, fasting C-peptide level drop was significantly smaller in Group 1 compared to Group 2 (30.6% and 47.5% respectively; p=0.001). Likewise, daily insulin doses decreased significantly in both groups (-0.59±0.14units/kg and -0.37±0.24units/kg respectively; p=0.001). All patients recruited completed the study. No adverse events were reported.

**Conclusion:**

Combined therapy with cholecalciferol and lansoprazole for six months was associated with smaller decline in residual β-cell function and lower insulin requirements in children with new-onset type 1 diabetes. Preliminary findings of this small-scale study need to be confirmed by larger studies.

**Registry of Clinical Trials:**

(www.ctri.nic.in) under number REF/2021/03/041415 N.

## INTRODUCTION

Type 1 diabetes (T1D) is the most common endocrine disorder in children. In T1D, autoimmune destruction of pancreatic β-cells leads to insulin deficiency. Affected patients usually retain only 10%-30% of their β-cell mass at the time of disease onset.^(1)^ Residual β-cell function (RBCF) is also almost completely lost within a variable time frame after T1D diagnosis due to persistent autoimmune destruction.^(2)^ However, RBCF preservation is important for better long-term outcomes in T1D. Even marginal RBCF protection is associated with clinically significant benefits.^(3)^ Patients with some endogenous insulin secretion have a lower risk of severe hypoglycemia and better glycemic control in the long term.^(4)^ In the Diabetes Control and Complications Trial, even modest levels of β-cell activity upon enrollment were associated with lesser blood glucose variability and lower incidence of retinopathy and nephropathy.^(5)^ The protective effect of RBCF against retinopathy may last for more than ten years.^(6)^ Residual β-cell function also has a direct favorable impact on physical growth in children with T1D, regardless of glycemic control quality.^(7)^ Preservation of RBCF is therefore desirable in patients with T1D.

It is commonly thought that enhanced glycemic control preserves RBCF.^(5,8)^ However, a recent systematic review and meta-analyses failed to generate robust evidence concerning the ability of interventions aimed at improving glycemic control to preserve RBCF in new-onset T1D, particularly randomized controlled trials.^(9)^ In the past few decades, several immunotherapeutic interventions designed to preserve RBCF have been tested, such as anti-CD antibodies (Teplizumab, Otelixizumab and Rituximab), CTLA4-Ig (Abatacept), tumor necrosis factor-alpha (TNF-α) inhibitor (Etanercept), IL-1 receptor antagonist (Anakinra), and intralymphatic injection of GAD65, a vaccine formulated with aluminum hydroxide (GAD-alum).^(10)^ Still, immunotherapeutic approaches have achieved limited long-term success in RBCF preservation, primarily due to the significant side effects of immunosuppressive drugs.^(10)^ Regenerative strategies to maintain RBCF, especially DYRK1A inhibitors, seem promising. However, these therapies are still in the experimental phase.^(11)^ Recent and ongoing research into RBCF preservation appears to point towards combination therapy with immunosuppressive agents and relatively non-toxic immunomodulatory molecules such as vitamin D.^(10,12)^ In these circumstances, less toxic, readily available, affordable long-term interventions aimed at RBCF preservation are worthy of investigation.^(10)^

Use of vitamin D supplementation to preserve RBCF is controversial. Enhanced insulin production and sensitivity in response to vitamin D supplementation has been demonstrated in pre-clinical research. However, human studies with T1D patients yielded variable results.^(12–14)^ Fortunately, the protective effect of vitamin D supplementation on RBCF is supported by studies with patients with modest C-peptide levels treated with high doses of cholecalciferol for longer periods of time.^(15–17)^ The immunomodulatory effects of 25-hydroxy-vitamin D [25-(OH)D] appear to arise at serum concentrations between 40 and 60ng/mL.^(14,15)^ Another class of drugs with potential effects on pancreatic β-cells and glucose metabolism are proton pump inhibitors (PPIs), such as pantoprazole and lansoprazole.^(18)^ These safe medications are widely used in treatment of acid-related gastric disorders and have been shown to increase gastrin levels. In turn, gastrin plays an important role in β-cell neogenesis regulation.^(19)^ Improved glycemic control in response to PPIs has been reported in experimental and clinical studies.^(20,21)^ However, the effects of PPIs on RBCF preservation in patients with type 2 diabetes are inconsistent.^(22,23)^ In patients with T1D, combination therapy with lansoprazole and sitagliptin did not show protective effects on RBCF. Nevertheless, authors of that study suggested preliminary results could be used to inform future studies with a larger number of patients, in particular patients with high C-peptide levels at baseline.^(24)^ Given the effects of PPIs on β-cell neogenesis, these agents are good candidates for combination therapy with immunomodulatory drugs. The goal of immunomodulatory therapy is to reduce the intensity of the ongoing autoimmune destruction of β-cells, ultimately leading to RBCF preservation.

## OBJECTIVE

To investigate the effects of combination therapy with cholecalciferol and lansoprazole on residual β-cell function and glycemic control in children with new-onset type 1 diabetes.

## METHODS

This study was carried out in compliance with the current World Medical Association guidelines (revised Declaration of Helsinki) and approved by the Institutional Ethics Committee (INT/IEC/2020/SPL-1366, reference # NK/6666/MD/4150). Children agreed to participate. A written informed consent form for voluntary participation was signed by their parents. This study was performed at a tertiary care pediatric teaching hospital located in Northwest India, between January and December 2021.

### Study subjects

Children aged 6-12 years and newly diagnosed with T1D were recruited at admission for inpatient diabetes education or at the first follow-up appointment after diagnosis. Diabetes diagnosis was based on International Society for Pediatric and Adolescent Diabetes criteria 2018.^(25)^ Additional criteria included positivity for at least one of the three routinely measured pancreatic autoantibodies (anti-glutamic acid decarboxylase 65/GAD65), anti-insulin/IAA) and anti-islet cell/ICA) and fasting C-peptide levels ≥0.5ng/mL. Exclusion criteria were presence of other comorbidities, such as celiac disease, thyroid dysfunction, hepatic or renal disease, and vitamin D deficiency (serum 25-(OH)D concentrations <20ng/mL). Age and sex-matched children with T1D who also met inclusion and exclusion criteria were recruited as controls. Willingness to participate was assessed upon enrollment.

### Intervention

Patients were maintained on their respective insulin regimens and kept records of self-monitored blood Glucose (SMBG) and insulin doses taken. Caregivers were instructed to self-titrate insulin doses to keep blood glucose levels within target ranges for age. A computer-generated random number table was used to allocate patients to the intervention (Group 1) or the control (Group 2) group. Group 1 children received 2,000IU of cholecalciferol and 15mg (<30kg) or 30mg (>30kg) of lansoprazole per day. Lansoprazole doses used in this study are the standard recommended doses for children and were similar to doses used in a prior T1D study.^(24)^ Controls children did not receive supplementation.

### Follow-up

Children were followed at three-month intervals for six months. Follow-up assessments were carried out by the same physicians (RR and DD) and the same instructions were provided throughout the follow-up period. At every appointment, children were submitted to a thorough physical examination and anthropometric measurements. Diabetes-related events such as ketoacidosis and hypoglycemic episodes, and potentially drug-related new symptoms were also recorded. Self-monitoring of blood glucose was carried out 3-4 times daily (fasting, before lunch and dinner, and at bedtime). Averaged SMBG readings from seven consecutive days prior to follow-up visits were used to calculate mean fasting blood glucose and mean blood glucose levels. Likewise, daily insulin doses taken in the last seven days were averaged to obtain mean daily insulin doses.

Serum 25-(OH)D, HbA1c and fasting C-peptide levels were measured at all time points (baseline, three-month and six-month follow-up). At every follow-up visit, vitamin D toxicity and lansoprazole-related side effects were investigated by measuring serum calcium levels and urinary calcium:creatinine ratio, and serum bilirubin, alanine aminotransferase (ALT) and aspartate aminotransferase (AST) levels respectively.

### Laboratory methods

Plasma C-peptide levels were measured using a sandwich-type Electrochemiluminescence Immunoassay (ECLIA) run on a fully automated analyzer (E 601, Roche Diagnostics GmbH, Mannheim, Germany) with reagent kits and calibrators provided by the same manufacturer. The tri-level Lyphocheck Immunoassay Plus (Bio-Rad laboratories, Irvine, CA) was used as control. The detection limit of this method is 0.01ng/mL; intra-assay and inter-assay coefficients of variations (CVs) are 4.6% and 5.0% respectively. Hemoglobin A1c levels were estimated using an automated high-performance liquid chromatography system based on ion exchange resins, with appropriate control and calibrators supplied by the same manufacturer (D-10, BioRad, USA). Serum 25-(OH)D concentrations were measured by competitive ECLIA on a fully automated analyzer (E 601), using kits and calibrators provided by the same manufacturer.

### Outcome measures

The primary outcome was change in fasting C-peptide levels. Secondary outcomes were changes in HbA1c levels, mean daily insulin dose, mean fasting blood glucose, and mean blood glucose levels from baseline to the end of the study.

### Statistical analysis

Descriptive statistics were used to describe different variables. Quantitative variables were reported as mean and standard deviation (SD) or median and interquartile range (IQR). Qualitative variables were described as proportions. The Student’s *@t*-test or variance analysis was used to compare means and mean changes in efficacy variables between groups. Mean absolute and percentage changes in fasting C-peptide levels from baseline to end of study were calculated for both groups and compared using the Student’s *t*-test. The χ^2^ test was used to examine associations between categorical variables. Statistical tests were two-sided, and the level of significance was set at 5%.

## RESULTS

The number of children admitted with T1D during the experimental period totaled 111. Of these, 56 were newly diagnosed. Twenty-eight children were eligible for enrollment. Reasons for exclusion were age under six years (23 patients), other autoimmune diseases (1 patient), low fasting C-peptide levels (2 patients) and failure to adhere to the protocol (2 patients). Group 1 children were slightly older than Group 2 children (mean age 8.6±2.1 and 7.2±1.2 years respectively; p=0.04). Each group had seven boys. Five children in each group had presented in diabetic ketoacidosis. The mean number of days from T1D diagnosis to recruitment was 17.2±14.5 (23.0±17.3 and 11.5±8.1 days, Group 1 and Group 2 respectively; range 2-60 days). All patients were on a basal-bolus insulin regimen with insulin Glargine (basal) and insulin Aspart or Lispro (bolus).

### Comparative analysis of primary efficacy parameters after intervention

Mean fasting blood glucose remained unchanged from baseline to end of study in both groups. However, Group 1 children had lower mean fasting blood glucose values at the three-month follow-up (Table 1). Mean blood glucose values were also significantly lower in Group 1 children at six-month follow-up. In contrast, mean HbA1c values remained unchanged throughout the experimental period in both groups. The mean daily insulin dose was significantly lower in the Intervention Group (Group 1) at the end of the study (Table 1). Mean fasting C-peptide levels did not differ from baseline to end of study in any of the groups.

**Table 1:** Comparison of treatment efficacy variables in both study groups at different time points

Variable	Timepoints	Group 1 (n=14) Mean±SD	Group 2 (n=14) Mean±SD	p value
HbA1c (%)	Baseline	12.8±2.5	13.2±2.8	0.65
	3 months	7.8±1.3	7.6±1.2	0.69
	6 months	7.4±1.3	7.3±1.2	0.83
Fasting blood glucose (mg/dL)	Baseline	114.5±19.9	119.4±41.7	0.69
	3 months	115.6±30.8	138.1±24.6	0.04
	6 months	114.9±34.2	128.1±26.1	0.26
Mean blood glucose (mg/dL)	Baseline	141.6±36.9	161.7±29.8	0.12
	3 months	134.4±21.4	151.0±27.8	0.08
	6 months	131.0±26.6	157.8±33.8	0.02
Daily insulin dose (units/kg)	Baseline	1.14±0.43	1.27±0.69	0.58
	3 months	0.58±0.34	0.82±0.55	0.18
	6 months	0.55±0.28	0.89±0.59	0.06
Fasting C-peptide (ng/mL)	Baseline	0.62±0.21	0.61±0.19	0.85
	3 months	0.64±0.51	0.42±0.21	0.15
	6 months	0.43±0.26	0.32±0.23	0.26

SD: standard deviation.

Comparative analysis of mean changes in primary efficacy parameters from baseline to end of study revealed significant differences in both groups. Mean fasting C-peptide levels dropped significantly (mean drop -0.19±0.09ng/mL, p=0.04 and -0.28±0.08ng/mL, p=0.001, Group 1 and in Group 2 respectively). However, C-peptide level drop was significantly smaller in Group 1 relative to Group 2 (30.6% and 47.5%; p=0.001) (Table 2). Likewise, mean daily insulin dose decrease differed significantly between groups (-0.59±0.14units/kg and -0.37±0.24units/kg, Group 1 and Group 2 respectively; p=0.001). The decrease in mean fasting blood glucose levels was significantly greater in Group 1 relative to Group 2. However, mean blood glucose and HbA1c level changes were similar in both groups (Table 2).

**Table 2:** Comparison of mean changes in efficacy parameters from baseline to end of study

Variable	Group 1 Mean±SD	Group 2 Mean±SD	p value
Daily insulin dose (U/kg/d)	-0.59±0.14	-0.37±0.24	0.001
HbA1c (%)	-5.3±0.77	-5.8±0.83	0.07
Fasting blood glucose (mg/dL)	0.4±10.5	8.7±13.17	0.07
Mean blood glucose (mg/dL)	-10.5±12.17	-3.9±12.07	0.16
Fasting C-peptide (ng/mL)	-0.19±0.09	-0.28±0.08	0.001

SD: standard deviation.

### Monitoring of drug-related side effects and adherence to study protocol

Baseline mean total 25-(OH)D concentrations were similar in both groups. However, mean total 25-(OH)D concentrations were significantly higher in Group 1 at the end of the study. Mean serum calcium concentrations and urinary calcium: creatinine ratio remained unchanged in both groups. Likewise, additional variables did not differ over the course of the experimental period (Table 3). Adherence to the experimental protocol was assessed by repeated verbal inquiries. All patients recruited completed the study. No adverse events or recurrence of diabetic ketoacidosis were reported.

**Table 3:** Comparison of clinical and laboratory variables monitored in both groups during the study

Variable	Timepoints	Group 1 (n=14) Mean±SD	Group 2 (n=14) Mean±SD	p value
Body mass index (kg/m^2^)	Baseline	13.9±2.8	15.4±4.0	0.26
	3 months	14.5±2.9	15.4±2.2	0.38
	6 months	14.8±2.5	15.9±2.1	0.21
Serum 25(OH)D (ng/mL)	Baseline	43.8±10.5	49.0±13.9	0.26
	3 months	41.6±8.2	39.2±10.2	0.48
	6 months	39.0±8.9	31.8±10.3	0.05
Serum calcium (mg/dL)	Baseline	9.2±0.3	9.3±0.75	0.89
	3 months	9.2±0.3	9.4±0.3	0.11
	6 months	9.4±0.2	9.6±0.6	0.35
Alanine aminotransferase (IU/L)	Baseline	24.9±10.8	21.0±6.6	0.26
	3 months	27.4±14.7	22.6±9.1	0.31
	6 months	29.3±14.6	29.0±25.6	0.96
Aspartate aminotransferase (IU/L)	Baseline	28.5±11.9	25.4±8.6	0.44
	3 months	30.5±8.8	26.0±8.9	0.19
	6 months	33.0±18.7	26.4±12.4	0.28
Serum total bilirubin (mg/dL)	Baseline	0.4±0.15	0.9±1.6	0.22
	3 months	0.4±0.18	0.9±1.8	0.27
	6 months	0.4±0.09	1.0±1.9	0.26
Serum albumin (mg/dL)	Baseline	4.0±0.56	4.0±0.7	0.99
	3 months	3.9±0.4	3.9±0.7	0.79
	6 months	3.9±0.5	4.1±0.9	0.69
Urine calcium:creatinine ratio	Baseline	0.14±0.1	0.17±0.0	0.48
	3 months	0.12±0.0	0.16±0.0	0.19
	6 months	0.13±0.0	0.15±0.0	0.65

SD: standard deviation.

## DISCUSSION

In this study combined use of cholecalciferol and lansoprazole was associated with slower RBCF decline in children with new-onset T1D. Importantly, effects on RBCF were obtained in a relatively short period of time (six months). In a prior study with a similar cohort of children with T1D treated exclusively with cholecalciferol at the same dose for a similar period, only a trend towards slower RBCF decline was observed in the intervention compared to Control Group.^(16)^ Cholecalciferol supplementation must be maintained for at least one year to elicit appreciable effects on RBCF.^(15,17)^ Hence, additional protective effects on RBCF in this study may have been due to lansoprazole. In the REPAIR-T1D trial, a weak trend towards RBCF preservation was noted in a subgroup of patients with elevated glucagon-like peptide 1 (GLP-1) and gastrin concentrations, suggesting indirect effects of lansoprazole.^(24)^ Therefore, combination therapy may have protected RBCF via direct effects of lansoprazole on β-cells neogenesis and modulation of immunological mechanisms of β-cell destruction by cholecalciferol.^(14,18)^

Another important finding was the lower insulin requirement in the Intervention Group despite similar glycemic parameters in both groups. Insulin requirements decreased in both groups, suggesting some children may have achieved partial remission. Partial remission shortly after diagnosis is common in children with T1D. In these cases, insulin requirements decrease due to sufficient insulin production by remaining β-cells. Also, recovery of immunological tolerance to β-cell auto-antigens may allow partial β-cell regeneration.^(26)^ Given the small sample size, lower insulin requirements in Group 1 may have reflected a larger number of children experiencing the partial remission in this study. However, lower insulin requirements may also have been due to enhanced RBCF protection and resultant higher endogenous insulin production in the Intervention Group. As in several past interventional therapy studies,^(24,27)^ patients with new-onset T1D in this sample were recruited while they still had sufficient RBCF to enable preservation. In prior studies, fasting C-peptide concentrations >0.5ng/mL were deemed sufficiently high upon enrollment.^(15,17)^ Low baseline C-peptide concentration was one of the potential reasons for negligible RBCF protection reported in prior vitamin D supplementation studies.^(28,29)^

Preliminary findings of this study suggest combination therapy with cholecalciferol and lansoprazole has protective effects on RBCF. Such protective effects play an important role in face of the need to preserve RBCF at relatively low costs and with less toxic interventions. Significant progress has been made in our understanding of the complex immunopathophysiology of β-cell destruction. Still, successful RBCF preservation after clinical onset of T1D has not been achieved so far.^(30)^ There is clearly a need for synergistic agents targeting multiple biological pathways of immune response.^(31)^ Over the last few years, vitamin D has become an integral part of RBCF-preserving interventions owing to its well-studied immunomodulatory effects in T1D.^(13–17,32–34)^ As to lansoprazole, preliminary data on combination therapy with sitagliptin revealed a weak trend towards RBCF preservation in a subgroup of T1D patients with increased GLP-1 and gastrin concentrations.^(24)^ Findings of the 24-month reassessment of REPAIR-T1D trial patients shall further elucidate the role of lansoprazole in RBCF preservation.^(24)^ The theoretical risk that increased insulin production in response to RBCF-preserving effects of PPIs may further stimulate autoimmunity may be mitigated by combination therapy with immunomodulatory drugs, as suggested by REPAIR-T1D trial investigators. Hence, combined therapy with lansoprazole and cholecalciferol, as used in this trial, constitutes a disease-modifying treatment for T1D which warrants further investigation.

In this study, mean 25-(OH)D concentrations in the optimal range in both groups at enrollment can be explained by vitamin D supplementation prescription to all patients at admission. In Northwest India, vitamin D deficiency is still commonly detected in children with T1D at admission.^(35,36)^ Further decline in serum 25-(OH)D concentrations is expected after admission.^(37)^ Hence, children admitted with T1D receive at least one 60,000IU of cholecalciferol as standard of care at our hospital. In the Intervention Group, mean serum 25-(OH)D concentrations remained within optimal ranges throughout the study. Optimal serum 25-(OH)D concentrations are thought to be essential to elicit immunomodulatory effects.^(38)^

This study has several limitations. Firstly, it was designed as a pilot study due to the age range of the study population. Also, an initial sample size of 20 children per group was defined based on convenience and practical considerations, but only 14 patients per group could be recruited due to the prevailing COVID-19 situation.^(39)^ Secondly, potential immunological effects of intermediary hormones such as gastrin, which seem to mediate the action of selected drugs, were not examined and their concentrations were not estimated.^(24)^ Thirdly, unblinded design may have introduced an investigator bias.^(40)^ Lastly, partial remission cannot be fully confirmed in a short six-month follow-up. Despite these limitations, findings of this study constitute important preliminary data to inform more extensive research.

## CONCLUSION

In conclusion, combination therapy with cholecalciferol and lansoprazole for six months was associated with a slower decline in residual β-cell function and lower insulin requirements in children with new-onset type 1 diabetes. These findings are preliminary and must be confirmed by larger studies.
